# Knockdown of circSOD2 ameliorates osteoarthritis progression via the miR-224-5p/PRDX3 axis

**DOI:** 10.1186/s13018-023-03880-9

**Published:** 2023-06-13

**Authors:** Hao Li, Yong Cao, Chongfei Chang, Wenping Huang, Songchuan Su, Zhenggang Peng, Jiajin Zhang

**Affiliations:** 1 Department of Sports Medicine, Shenzhen Second People’s Hospital, Shenzhen, 518037 Guangdong China; 2 Department of Orthopaedic Emergency, Chongqing Orthopedic Hospital of Traditional Chinese Medicine, Chongqing, 400000 China; 3 Shenzhen Cheerland Danlun Biomedical Co. Ltd, Shenzhen, 518108 Guangdong China; 4 Department of Traumatic Orthopedics, Chongqing Orthopedic Hospital of Traditional Chinese Medicine, 9 Jiefang West Road, Yuzhong District, Chongqing, 400000 China

**Keywords:** circSOD2, miR-224-5p, PRDX3, OA

## Abstract

**Background:**

Although the implications of circular RNAs (circRNAs) with the progression of diverse pathological conditions have been reported, the circRNA players in osteoarthritis (OA) are barely studied.

**Methods:**

In this study, twenty-five OA patients who received arthroplasty were recruited for cartilage tissue collection. Public circRNA microarray data from Gene Expression Omnibus was retrieved for circRNA identification. An in vitro cell model of OA-related damages was constructed by treating human chondrocytes (CHON-001 cell line) with IL-1β, and circSOD2 siRNA was used to silence circSOD2 expression to study its functional role in apoptosis, inflammatory responses, and extracellular matrix (ECM) degradation. Besides, we investigated the functional interactions among circSOD2, miR-224-5p, and peroxiredoxin 3 (PRDX3) by luciferase reporter assay, RNA-immunoprecipitation assay, and quantitative reverse transcription polymerase chain reaction.

**Results:**

Our findings revealed the overexpression of circSOD2 in the OA cartilage and cell samples, and circSOD2 knockdown alleviated ECM degradation, inflammation, and apoptosis in CHON-001 cell model. In addition, our findings suggested the regulatory function of circSOD2 knockdown on miR-224-5p expression, while miR-224-5p was capable of downregulating PRDX3 expression. The co-transfection of miR-224-5p inhibitor or pcDNA-PRDX3 could prevent the effect of circSOD2 knockdown.

**Conclusion:**

Hence, our results demonstrated that knockdown of circSOD2 may serve as an intervention strategy to alleviate OA progression through modulating miR-224-5p/PRDX3 signaling axis.

**Supplementary Information:**

The online version contains supplementary material available at 10.1186/s13018-023-03880-9.

## Introduction

As one of the most prevalent chronic arthropathies, osteoarthritis (OA) is an endemic throughout the world. The progression of OA involves the degenerative alterations at the articular cartilage, which eventually results in sterile inflammation [1]. Apart from aging which is a leading risk factor, other causes of OA involve environmental factors, unhealthy lifestyle, and excessive exercise [2]. However, there remain limited therapeutic strategies for OA given its elusive etiology and pathogenesis [3]. The public health system is severely burdened by OA, which is linked to chronic pain, loss of functional mobility, and the decreased life quality of OA patients. Hence, the understanding of molecular players underlying the pathological progression of OA could provide insights into the therapeutic intervention.

Circular RNAs (CircRNAs) refer to a kind of non-coding RNAs whose structures are covalently closed and stable. Accumulating evidence pinpoints the critical roles of circRNAs in cellular functions through post-transcriptional regulation of gene expression in various disease progressions [4, 5]. Latest studies have also reported the pivotal actions of circRNAs on OA, indicating that their dysregulation in OA may be employed as diagnostic or therapeutic targets [6, 7]. The mechanistic studies of microRNA (miRNA) regulation by circRNAs have attracted growing interest in the field of RNA research. These studies highlighted that circRNAs act as molecular sponges of miRNA targets, thereby modulating the expression of downstream mRNAs in different cellular processes [8]. For instance, circRNA_0092516 depletion has been demonstrated to mitigate OA by promoting the proliferation of chondrocytes and impeding cellular apoptosis via miR-337-3p/PTEN pathway [9]. In addition, CircCDH13 was reported to contribute to the pathogenesis of OA through sponging miR-296-3p and regulating PTEN pathway [10]. In addition to circRNA, miRNAs have also been implicated in the pathological conditions of musculoskeletal tissues. Increasing evidence suggest that the dysregulation of miRNAs impinges on the progression of tendon injuries and osteoarthritis, which may serve as promising diagnostic and therapeutic biomarkers [11, 12].

In this study, the analysis of circRNA microarray data containing knee tissues of three normal subjects and three OA patients revealed the overexpression of hsa_circ_0004662 (circSOD2) in the OA patient samples, which was further validated in the cartilage samples from OA patients and healthy controls. It has been reported that circSOD2 promotes liver cancer cell proliferation and is implicated in cancer progression [13]. However, the functional engagement of circSOD2 in OA progression and the underlying mechanisms have yet to be clarified. Subsequent bioinformatic prediction and experimental validation revealed miR-224-5p as a downstream target of circSOD2. This finding is line with the previous observation that miR-224-5p expression was decreased in OA [14, 15]. We further confirmed PRDX3 as a target mRNA of miR-224-5p. Similarly, Xu et al. found that PRDX3 was overexpressed in OA samples [16]. In this study, our functional experiments further clarified that downregulating circSOD2 could alleviate OA-related damages in human chondrocyte cell model through targeting miR-224-5p/PRDX3 axis. The present work uncovered a novel regulatory module in OA progression and suggested circSOD2 as a novel molecular target for OA intervention.

## Methods

### Patients and tissue samples

All the patients signed an informed consent in a written form, and the present research work was approved by the Ethics Committee of Chongqing orthopedic hospital of traditional Chinese Medicine. The cartilage tissues from the knee of 25 OA patients who had undergone arthroplasty were collected and snap-frozen in liquid nitrogen for further analysis. Proteoglycan variations were assayed by Safranin O-F green staining, and further evaluation of histological data was accomplished through Osteoarthritis Research Society International (OARSI) scoring as previously reported, and Kellgren–Lawrence (K-L) grade was used to assess the cartilaginous structural degeneration in the patients based on arthroscopic analysis [17]. Normal articular cartilage tissues were isolated from the knee joints of 25 trauma patients without the diagnosis of OA as controls.

### Bioinformatics analysis

CircRNA expression profiling data (GSE175959 microarray analysis) was downloaded from the Gene Expression Omnibus (GEO) database (https://www.ncbi.nlm.nih.gov/geo/), which contains knee cartilage samples from three OA patients and three healthy controls. We screened the differentially expressed circRNAs under the following criteria: Log 2 (Fold Change) greater than 1 or less than − 1; and adj *p* value less than 0.05. Thereafter, we predicted the downstream miRNA candidates of hsa_circ_0004662 (circSOD2) via circBank (http://www.circbank.cn/), circinteractome (https://circinteractome.nia.nih.gov/) and starBase ver. 2.0 ( https://starbase.sysu.edu.cn). The mRNA targets of miR-224-5p as well as their bindings sites were further analyzed using starBase ver. 2.0.

### Cell lines and culture

We obtained human chondrocytes (CHON-001 cell line) from the CAS Cell Bank of Type Culture Collection (Shanghai, China). After heat inactivation under 37 °C in a 5% CO_2_ incubator, RPMI-1640 medium (HyClone, USA) was used to culture these cells, whose heat-inactivated FBS (Merck Millipore, Germany) content was 10% (vol/vol). The in vitro mimicking of OA-induced cell damages was established by treating CHON-001 cells with IL-1β (10 ng/mL; Sigma-Aldrich) for 48 h as per prior procedure [2, 18].

### qRT-PCR (quantitative reverse transcription polymerase chain reaction)

The Trizol reagent (Invitrogen, USA) was utilized for the extraction of total RNA from the cell culture, followed by synthesis of first strand cDNA with the first-stand cDNA synthesis Kit (Thermoscript, USA). For miRNA analysis, the cDNA synthesis was conducted using Mir-X miRNA first-strand synthesis kit (Takara, Otsu, Japan) following the manufacturer’s protocol. The resulted cDNA was then analyzed using the step-one plus qRT-PCR System (ABI Prizm, Applied Biosystems, USA) on the QuantStudio PCR system (BioRad, USA). For the analysis of relative gene expression, the 2^−△△Ct^ method was employed, with GAPDH as the internal reference for circSOD2/PRDX3 and U6 snRNA as the internal reference for the miRNA. The following primer sequences (Sangon Biotech, Shanghai) were used in qRT-PCR analysis: circSOD2 forward: 5′-AAACCACGATCGTTATGCTG-3′ and reverse: 5′-CGTTAGGGCTGAGGTTTGTC-3′; hsa-miR-224-5p forward: 5′-CAAGTCACTAGTGGTTCCGT TTAG-3′ and reverse: 5′-CTCAACTGGTGTCGTGGAGTC-3′; PRDX3 forward: 5′-TCGCAGTC TCAGTGGATTCC-3′ and reverse: 5′-ACAGCACACCGTAGTCTCGG-3′; hsa-miR-532-3p forward: 5′-ATCCTCCCACACCCAAGG-3′ and reverse: 5′-GTGCAGGGTCCGAGGT-3′; U6: forward, 5′-CTCGCTTCGGCAGCACAT-3′ and reverse 5′-AACGCTTCACGAATTTGCGT-3′; GAPDH: forward: 5′-GGTGAAGGTCGGAGTC-3′ and reverse: 5′-GAAGATGGTGATGGGATTTC-3′.

### Nuclear and cytoplasmic fractioning

For nucleoplasm fraction experiment, the nuclear and cytoplasmic faction was extracted using NE-PERNuclear and Cytoplasmic Extraction Reagents (Thermo Fisher Scientific, USA). Briefly, 1 × 10^6^ cells were harvested with trypsin–EDTA and then centrifuge at 500×*g* for 5 min. The cell pellet was washed once with PBS, and then re-suspended in 1-mL ice-cold CER I buffer and incubated on ice for 10 min. Then, the cells were mixed with 55-μL ice-cold CER II buffer for 1-min incubation, followed centrifugation at 16,000×*g* for 5 min. The resulted supernatant (cytoplasmic fraction) was collected in a fresh microvial, and the nuclear pellet was re-dissolved in 500-μL ice-cold NER buffer to obtain the nuclear soluble fraction. The total RNA in each fraction was purified using Trizol reagent (Invitrogen, USA) according to the manufacturer's protocol. An equal number of cells was used for total cell lysate RNA extraction, which served as the total cellular RNA level control for normalization. The extracted RNA samples were quantified by qRT-PCR.

### Transfection

CHON-001 cells were transfected with 1000 ng/mL circSOD2 siRNA, scramble siRNA, miRNA-NC or miRNA mimic (Hanheng, China) using Lipofectamine 20000 transfection reagent (Invitrogen, USA) as per the protocol of manufacturer. Cells were inoculated in a 6-well plate at the density of 5 × 10^5^ cells/well. 24 h after seeding, siRNA molecules or miRNA mimic was added into 100-µl Opti-MEM® I Reduced-Serum Medium (Invitrogen, USA), and then mixed with 6-µL Lipofectamine 2000 reagent. The mixture was incubated for 15 min before adding to the cells. The circSOD2 siRNA target sequence was 5′-ACGATCGTTATGCTGAGAGAT-3′.

### CCK-8 assay

The proliferative ability of cells was examined by a CCK-8 assay kit (Beyotime biotechnology, China) after being treated with the IL-1β and siRNA transfection. Cells inoculated in 96-well plates were cultured for 3 days, and at indicated time point, 15-μL CCK-8 reaction solution was added to the cell culture for 2-h incubation at 37 °C. Then, the medium was removed, and the cells were dissolved in 150 μL DMSO for 15 min. A microplate reader (Beyotime biotechnology, China) was adopted for determining the absorbance (OD450) in each well.

### Flow cytometry analysis of apoptosis

Initially, 5 × 10^5^ cells were harvested by trypsin and then suspended in 1-mL PBS (Phosphate-buffered saline). Next, 5 μL of Annexin-V FITC and propidium iodide staining reagent (Best-bio, China) was mixed with cell suspension for 15 min staining in the dark. After washing, the stained cells were re-suspended in 500-μL staining buffer, and the cell apoptosis ratio was analyzed by FACS Calibur flow cytometer (BD Biosciences, USA).

### Western blot

Total protein extracts were separated through SDS–PAGE (sodium dodecyl sulfate–polyacrylamide gel electrophoresis) and subsequently transferred onto PVDF (polyvinylidene difluoride) membranes, followed by blocking with 5% non-fat milk in TBS Tween-20 buffer (TBST) for 30 min. The membranes were proceeded with staining using primary antibodies including: Aggrecan (1:1000, ab3778, Abcam), MMP13 (1:1000, ab39012, Abcam), Collagen II (1:1000, ab34712, Abcam), PRDX3 (1:1000, ab73349, Abcam), and β-actin (1:1000, ab8226, Abcam). After washing with TBST, corresponding secondary antibody with HRP label was applied to detect the primary antibodies. For assessment of protein bands, an ECL chemiluminescence kit (Thermo Fisher Scientific, USA) was used for signal development, and β-actin was used for internal reference. The intensity of the blots was quantified by ImageJ (NIH, USA).

### Enzyme-linked immunosorbent assay (ELISA)

The ELISA Kits (RD, Minneapolis, USA) were utilized for assaying the concentrations of inflammatory cytokines including TNF-α, IL-6 and IL-8 level, as per the instructions of manufacturer. The cell media from different experimental groups were collected and subjected to a 10-min centrifugation (2000×*g*) for 10 min to collect the supernatant. 100-μL cellular supernatant medium was applied for measuring corresponding cytokine levels at 450 nm using a microplate reader (Bio-Rad). The concentration of each cytokine was determined based on the linear regression of the standards.

### RNA immunoprecipitation (RIP) assay

RIP assay was carried out using the RIP kit (Millipore, USA) as per the protocol of manufacturer. 1 × 10^6^ of CHON-001 cells were lysed using 1-mL IP lysis buffer (Beyotime biotechnology, China) on ice for 15 min. After centrifugation at 10,000×*g* for 10 min, 100 μL of supernatant was taken as the input, while the remaining supernatant was subjected to overnight incubation at 4 °C with 10 μg of Ago-2 (Argonaute-2) or IgG (Immunoglobulin G) (both from Abcam) antibody. Then, 100-μL streptavidin-magnetic beads were added to the solution for 1-h incubation at 4 °C. The sample was placed on the magnetic pedestal to collect the magnetic bead-protein complex. The co-precipitated RNA was purified using Trizol reagent (Invitrogen, USA), and qRT-PCR analysis was performed to detect the levels of miR-224-5p and circSOD2.

### RNA pull-down analysis

CHON-001 cells (1 × 10^6^) were lysed using 1 mL ice-cold IP lysis buffer for 15 min. After centrifugation at 10,000×*g* for 10 min, 100 μL of supernatant was taken as the input, while the remaining supernatant was subjected to overnight incubation with 50-nM biotin-labeled circSOD2 or control probe. The next day, 50-μL M-280 streptavidin beads (Sigma-Aldrich, Germany) were added to the solution for 1-h incubation at 4 °C. The sample was placed on the magnetic pedestal to collect the magnetic bead-protein complex. The magnetic beads were then harvested using a magnetic pedestal. The co-precipitated RNA was purified using Trizol reagent (Invitrogen, USA), and qRT-PCR analysis was performed to quantify the relative encirclement of miR-224-5p by circSOD2 or control probe.

### Dual-luciferase reporter assay

The construction of circSOD2/ PRDX3 wild-type reporter vectors (circCOL1A2-WT/PRDX3-WT) or circSOD2/ PRDX3 mutated reporter vectors (circSOD2-MUT/PRDX3-MUT) was accomplished by Sangon Biotech (Shanghai, China). Lipofectamine 2000 reagent was utilized for cellular transfection of the WT or MUT vectors into CHON-001 cells in the presence of miR-224-5p mimic or miR-NC. 8 h after transfection, the quantification and standardization of luciferase reporter activity were determined by a dual-luciferase reporter assay kit (Yeasen Biotech, China) on a luminescence microplate reader (BioRad, USA).

### Statistical analysis

Data were displayed as means ± SDs (standard deviations) from triplicate (at least) experiments. GraphPad Prism 5.0 software was utilized for performing statistical analyses. Two-group and multiple comparisons were conducted via the student’s T test or one-way ANOVA, respectively. Statistical significance was shown at *P* value < 0.05.

## Results

### CircSOD2 is overexpressed in the OA clinical samples and cell model

**Fig. 1:**
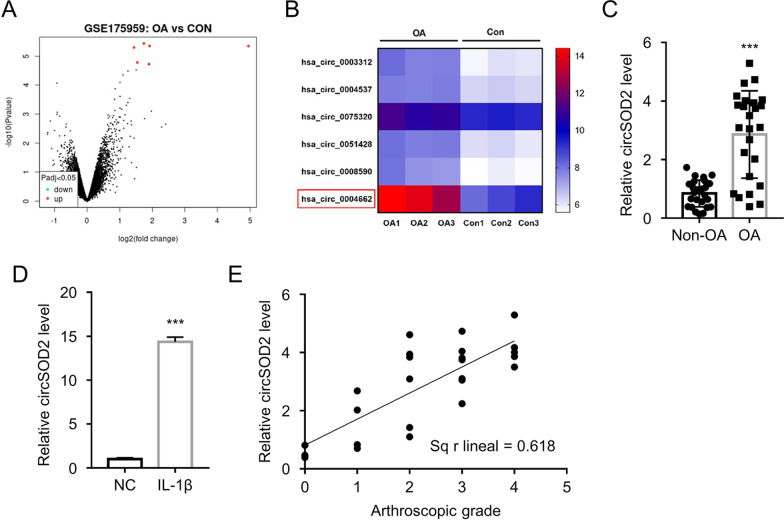
CircSOD2 is overexpressed in the OA clinical samples and cell model. **A** CircRNA expression profiling data (GSE175959) containing knee cartilage samples from three OA patients and three healthy controls. **B** The heatmap showing the top 6 overexpressed circRNAs in the OA samples. **C** qRT-PCR analysis of circSOD2 expression in the cartilage tissues of the knee joint from 25 OA patients who underwent arthroplasty and 25 controls. ***P < 0.001 in comparison with non-OA group. **D** qRT-PCR analysis of circSOD2 expression in CHON-001 human chondrocytes (CHON-001 cells) treated with or without inflammatory cytokine IL-1β. ***P < 0.001 in comparison with NC group. **E** The association of circSOD2 level with OA progression level. Arthroscopic analysis was performed in OA patients to assess cartilaginous degeneration based on Kellgren–Lawrence (K–L) scale (the higher the score, the more serious the degree of cartilage degeneration)

CircRNA expression profiling data (GSE175959) containing knee cartilage samples from three OA patients and three healthy controls were subjected to differential circRNA expression analysis. A total number of six significantly upregulated circRNAs in OA samples were identified using the threshold of Log_2_ Fold Change > 1 and adj *p* value < 0.05 (Fig. 1A, B). Among them, circ_0004662 (circSOD2) showed the strongest overexpression in OA samples. We also collected cartilage tissues of the knee joint from 25 OA patients who underwent arthroplasty and 25 healthy controls, and qRT-PCR analysis was performed to confirm the overexpression of the identified circRNAs. Consistently, expression of circ_0004662 (circSOD2) in OA patient tissues showed strongest overexpression than that in the controls (Fig. 1C). For other candidates, only circ_0051428 and circ_0008590 levels showed mild increase in OA samples (Additional file 1: Fig. S1). We therefore selected circSOD2 as our target for detailed investigation.

To enable to perform the mechanical study, an OA cellular model was developed by treating CHON-001 human chondrocytes (CHON-001 cell line) with inflammatory cytokine IL-1β. In comparison with the control cells, IL-1β challenge induced the elevated expression of circSOD2 (Fig. 1D), suggesting the implication of circSOD2 in the cellular damage model of OA. To further evaluate the association of circSOD2 level with OA progression, the arthroscopic analysis results of OA patients were graded based on Kellgren–Lawrence (K–L) scale to assess the cartilaginous structural degeneration (the higher the score, the more serious the degree of cartilage degeneration). The correlation analysis suggest that a higher expression level of circSOD2 is connected with a more serious cartilaginous degeneration in the patients (Fig. 1E). These results altogether indicate the involvement of circSOD2 upregulation in the progression of OA.

### CircSOD2 knockdown alleviates IL-1β-induced chondrocyte injury

**Fig. 2:**
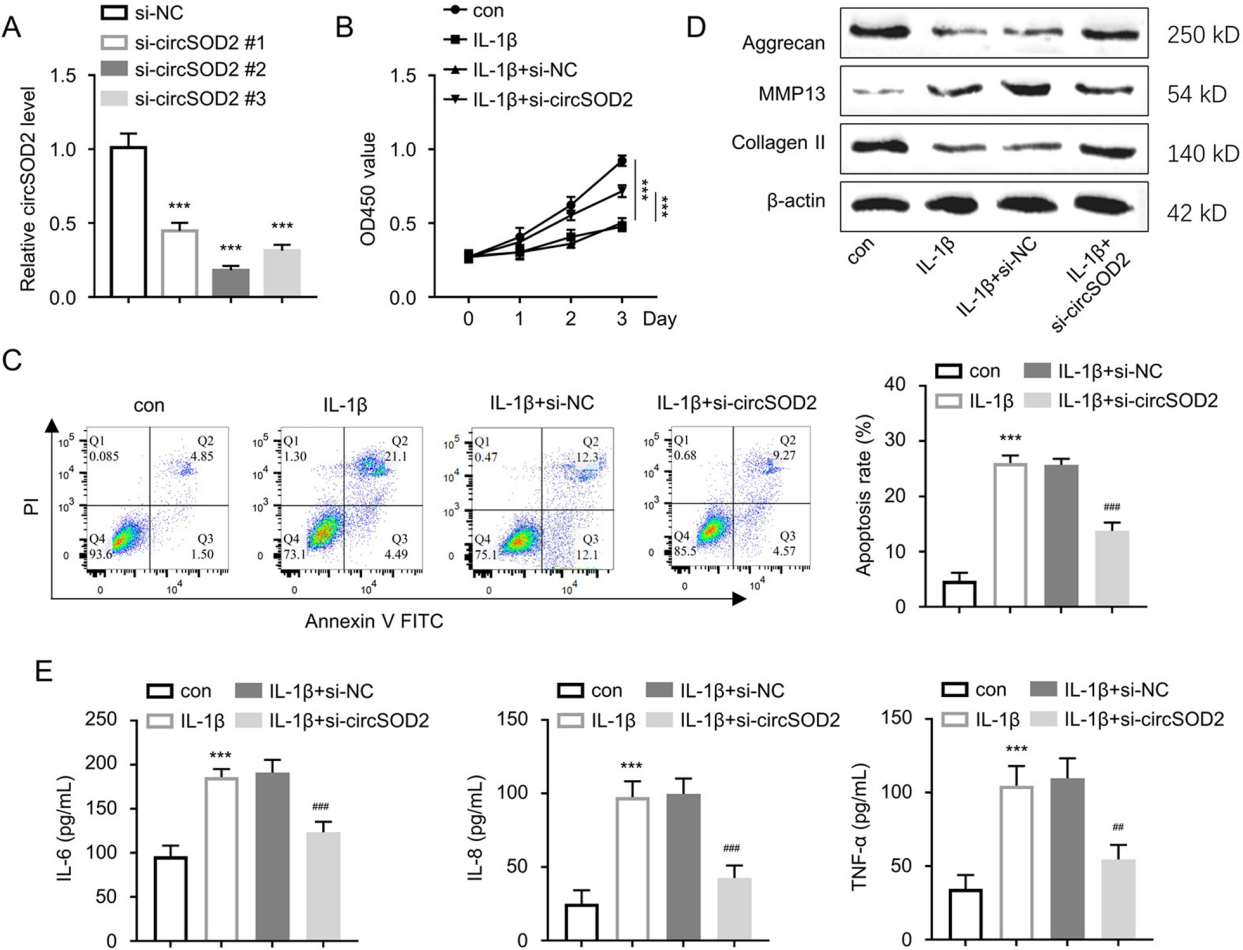
CircSOD2 knockdown alleviates IL-1β induced chondrocytes injury. **A** CircSOD2 expression was evaluated by qRT-PCR upon the transfection with control siRNA or siRNA targeting circSOD2 in CHON-001 cells. ***P < 0.001 versus the si-NC group. **B** CCK-8 assay of cellular proliferation in CHON-001 cells upon the transfection with control siRNA or siRNA targeting circSOD2. ***P < 0.001 in comparison with control group. **C** Flow cytometric detection of apoptosis in CHON-001 cells upon the transfection with control siRNA or siRNA targeting circSOD2. ***P < 0.001 versus the control group; ^###^*P* < 0.001 versus the IL-1β group. **D** Western blotting findings of the Aggrecan, MMP-13 and Collagen II protein levels in indicated experimental groups. **E** ELISA-based analysis levels of IL-6, IL-8, and TNF-α levels. ***P < 0.001 in comparison with control group; ^###^*P* < 0.001, ^##^*P* < 0.01 in comparison with IL-1β group

In order to validate the functional engagement of circSOD2 in OA, we knocked down circSOD2 in CHON-001 cells using three siRNAs targeting circSOD2. The knockdown efficiency determination by qRT-PCR suggested that si-circSOD2#2 was the most effective si-RNA to reduce circSOD2 in CHON-001 cells (Fig. 2A), and it was named as si-circSOD2 in subsequent studies. IL-1β challenge significantly suppressed the cell proliferation in the CCK-8 assay, while the transfection of si-circSOD2 could rescue cell proliferation upon IL-1β challenge (Fig. 2B). In order to confirm the impact of circSOD2 knockdown on IL-1β-elicited apoptosis, we conducted flow cytometry analysis with Annexin V and PI staining. The results showed that that IL-1β-treatment induced cell apoptosis in CHON-001 cells, but si-circSOD2 transfection decreased the apoptosis rate (Fig. 2C).

Proteins associated with extracellular matrix (ECM) degradation in OA, including Aggrecan, MMP-13, and Collagen II [19, 20], were then analyzed by Western blot. The data showed that circSOD2 knockdown could mitigate the IL-1β-triggered ECM degradation in IL-1β-treated CHON-001 cells, since circSOD2 silencing promoted Aggrecan and Collagen II levels, but downregulated MMP-13 levels (Fig. 2D). Furthermore, ELISA was adopted to detect the inflammatory cytokines (IL-6, IL-8 and TNF-α) in the cell culture supernatant of CHON-001 cells. IL-1β induction caused the over-production of these cytokines, and circSOD2 knockdown significantly attenuated their production levels (Fig. 2E). These results suggest that circSOD2 knockdown could mitigate IL-1β-induced damages in chondrocyte model of OA.

### CircSOD2 is mainly localized in the cytoplasm and targets miR-224-5p

**Fig. 3:**
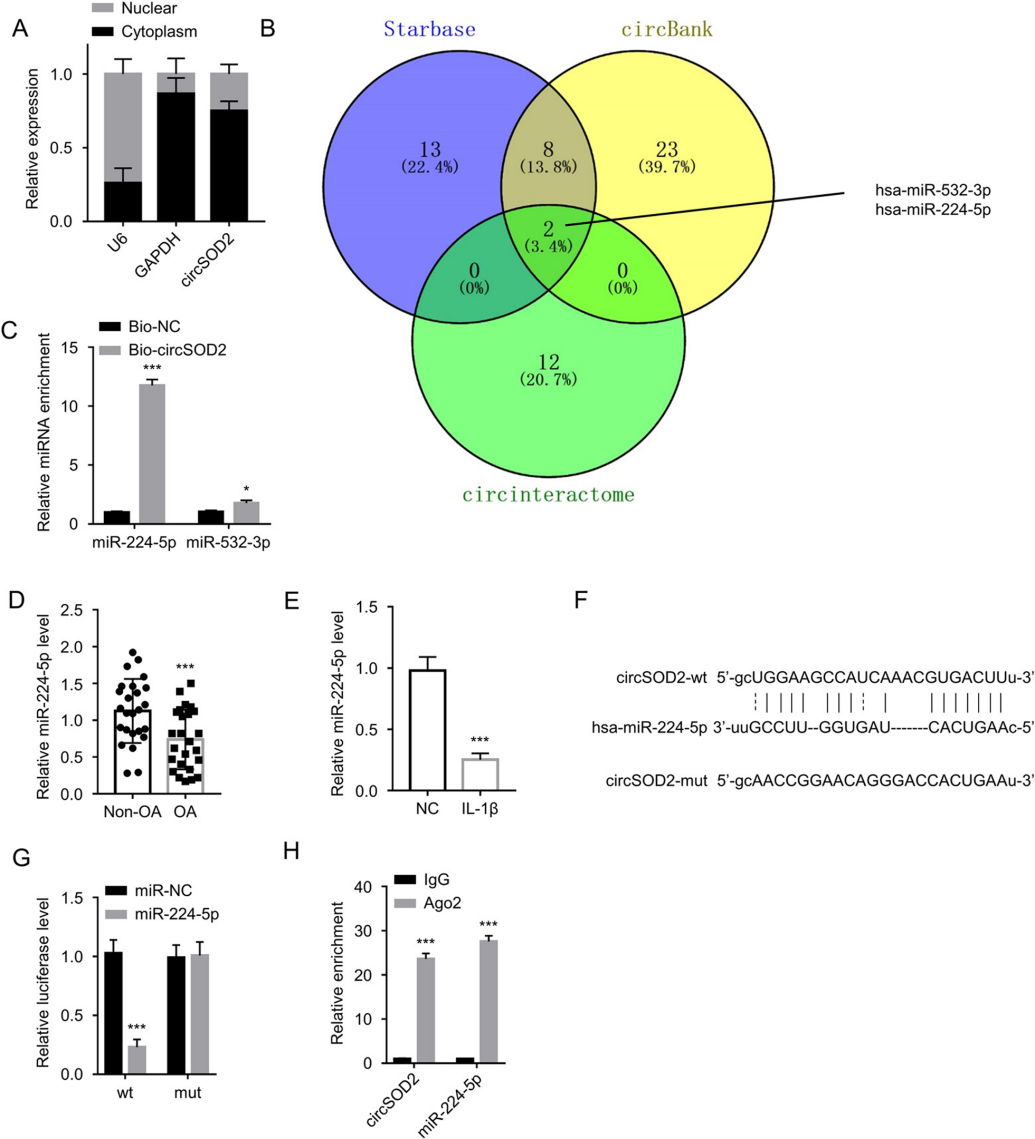
CircSOD2 is mainly localized in the cytoplasm and targets miR-224-5p. **A** CircSOD2 expression in nuclear and cytoplasmic fractions of CHON-001 cells was assessed by qRT-PCR. **B** miRNA candidates of circSOD2 were predicted using circBank, Starbase, and circinteractome databases. **C** RNA pull-down analysis using biotin-conjugated circSOD2 probe or control probe. The relative enrichment of miR-224-5p and miR-532-3p was determined by qRT-PCR. *P < 0.05, ***P < 0.001 versus the Bio-NC group. **D** qRT-PCR analysis of miR-224-5p expression in the cartilage samples from OA patients and controls. ***P < 0.001 compared to Non-OA group. **E** miR-224-5p expression in IL-1β-treated CHON-001 cells and the untreated cells. ***P < 0.001 compared to NC group. **F** StarBase predicted the binding sites between circSOD2 and miR-224-5p. **G** Dual-luciferase reporter assay in CHON-001 cells using circSOD2 WT or MUT reporter, in the presence of miR-224-5p mimic or miR-NC. ***P < 0.001 compared to miR-NC group. **H** RIP assay of circSOD2 and miR-224-5p using Ago2 or IgG isotype control. The relative enrichment of miR-224-5p and miR-532-3p was determined by qRT-PCR. ***P < 0.001 versus the IgG group

To clarify the regulatory mechanism of circSOD2 in chondrocyte cells, we first performed nuclear-cytoplasmic isolation experiment in CHON-001 cells to study the subcellular localization of circSOD2. qRT-PCR quantification revealed the predominant cytoplasmic localization of circSOD2 in CHON-001 cells (Fig. 3A). We then sought to seek for the miRNAs candidates of circSOD2. Through the predictions of circBank, Starbase, and circinteractome public databases, two potential target miRNAs (hsa-miR-532-3p and hsa-miR-224-5p) of circSOD2 were identified (Fig. 3B). To confirm their physical interaction with circSOD2, we then conducted RNA pull-down analysis using biotin-conjugated circSOD2 probe or control probe. miR-224-5p was heavily enriched by circSOD2 probe, while miR-532-3p was only marginally precipitated by circSOD2 (Fig. 3C); thus, miR-224-5p could be a downstream target absorbed by circSOD2.

We further analyzed miR-224-5p expression levels among the cartilage samples from OA patients and controls, revealing a reduced miR-224-5p expression in the OA group (Fig. 3D). Similarly, miR-224-5p expression in IL-1β-treated CHON-001 cells was repressed compared to that in the untreated cells (Fig. 3E). Besides, based on the binding sites prediction of circSOD2 and miR-224-5p through starBase (version 2.0) (Fig. 3F), we conducted luciferase reporter assay using wild-type (WT) binding site reporter vector or reporter with mutated (MUT) binding sequence. The overexpression of miR-224-5p using miRNA mimic suppressed the activity of WT reporter, but showed no effect on MUT reporter (Fig. 3G), suggesting the interaction between circSOD2 and miR-224-5p via predicted bindings sequences. Meanwhile, as revealed by the RIP assay, circSOD2 and miR-224-5p could be simultaneously enriched using Ago-2 antibody, which further corroborated their interplay in Ago-2 containing complex (Fig. 3H).

### miR-224-5p negatively regulates PRDX3 expression

**Fig. 4:**
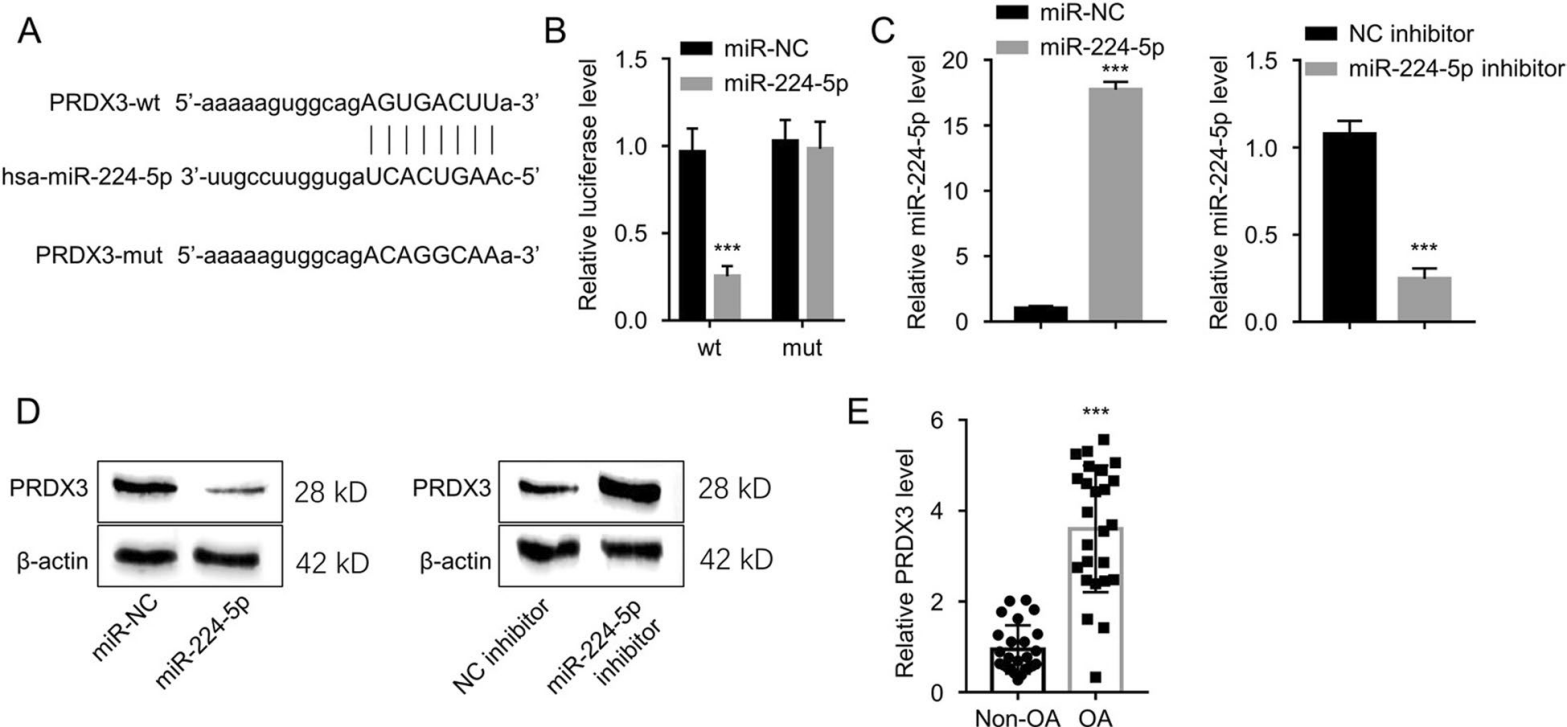
miR-224-5p targets PRDX3. **A** StarBase-aided prediction of the binding sites between miR-224-5p and PRDX3. **B** Dual-luciferase reporter assay in CHON-001 cells using PRDX3 WT or MUT reporter, in the presence of miR-224-5p mimic or miR-NC. ***P < 0.001 versus the miR-NC group. **C** qRT-PCR analysis of the miR-224-5p expression in CHON-001 cells upon transfection of miR-224-5p mimic or inhibitor. ***P < 0.001 versus the miR-NC or NC inhibitor group. **D** The protein level of PRDX3 was evaluated by Western blot in CHON-001 cells upon transfection of miR-224-5p mimic or inhibitor. **E** qRT-PCR analysis of PRDX3 expression in the cartilage samples from OA patients and controls. ***P < 0.001 versus the Non-OA group

Next, miR-224-5p sequence was subjected to starBase analysis for the purpose of exploring the mRNA targets. Multiple mRNA candidates were identified in starBase (Additional file 2: Fig. S2). The narrow down the most promising target, miR-224-5p mimic was transfected into CHON-001 cells, and qRT-PCR analysis showed only PRDX3 mRNA was significantly suppressed by miR-224-5p mimic (Additional file 2: Fig. S2). Further, the binding sites of miR-224-5p with PRDX3 mRNA was predicted by starBase (Fig. 4A), and dual-luciferase reporter assay was conducted using WT or MUT luciferase reporter. The administration of miR-224-5p mimic repressed the luciferase activity of PRDX3-WT reporter, while this effect was abolished in the MUT reporter (Fig. 4B), indicating the interplay of miR-224-5p with PRDX3. The transfection of miR-224-5p mimic increased the level of miR-224-5p and its inhibitor suppressed miR-224-5p expression (Fig. 4C). Next, we detected the PRDX3 protein level in CHON-001 cells upon the transfection of miR-224-5p mimic or inhibitor. The results showed that PRDX3 protein level was increased after miR-224-5p inhibitor transfection, while miR-224-5p mimic reduced PRDX3 protein level (Fig. 4D). Furthermore, the PRDX3 expression showed downregulation in 25 OA cartilage tissues in comparison with the control samples (Fig. 4E). Hence, PRDX3 is negatively targeted by miR-224-5p.

### CircSOD2 regulates IL-1β-induced chondrocyte injury via miR-224-5p/PRDX3 axis

**Fig. 5:**
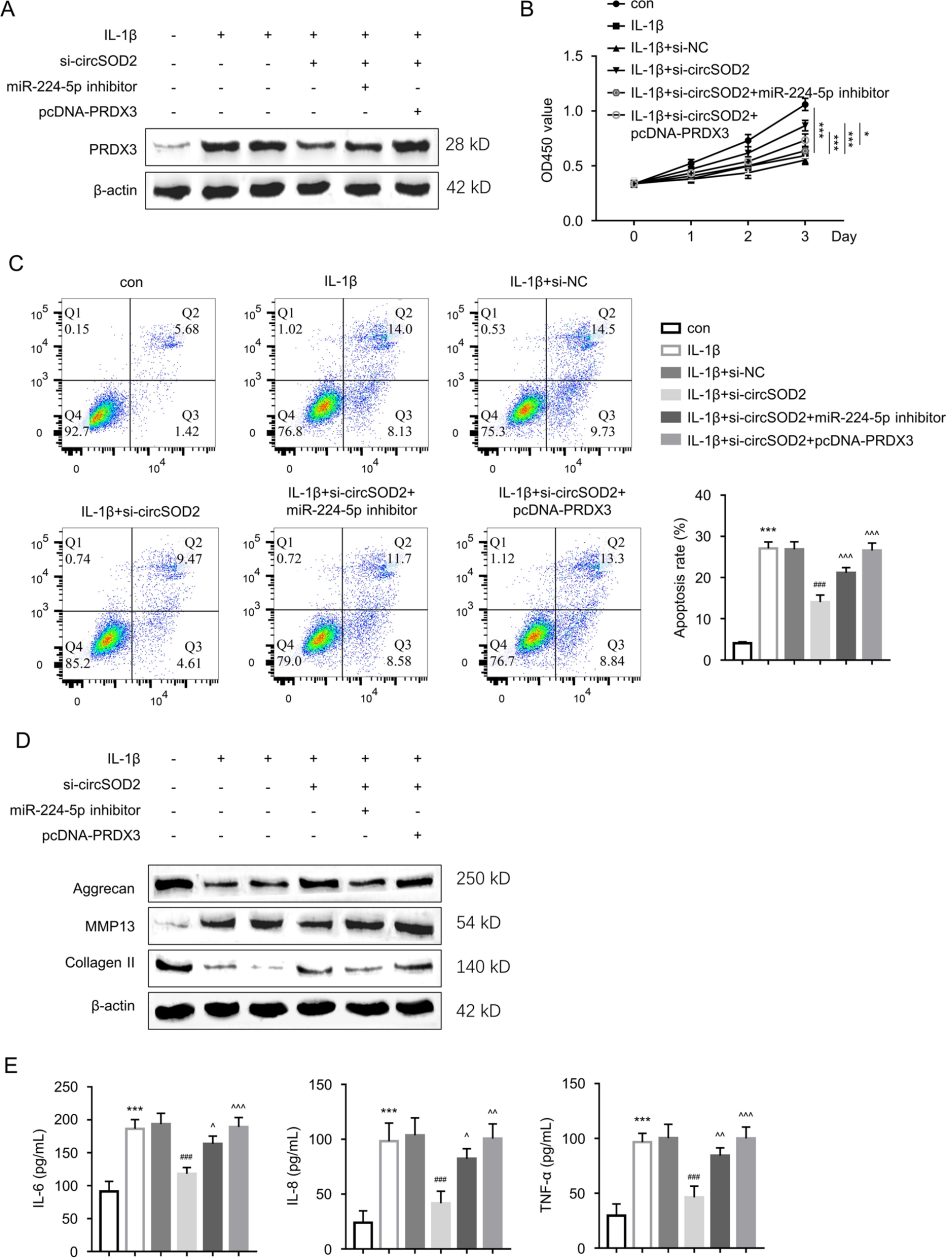
CircSOD2 regulates IL-1β induced chondrocyte injury through miR-224-5p/PRDX3 pathway. CHON-001 cells transfected with si-NC, si-circSOD2, si-circSOD2+miR-224-5p inhibitor or si-circSOD2+PRDX3 expression vector in the presence of IL-1β challenge. **A** Western blotting detection of the PRDX3 protein level in the above groups. **B** CCK-8 assay analysis of cellular proliferation in the above experimental groups. *P < 0.05, ***P < 0.001 compared to control group. **C** Flow cytometric detection of apoptosis in the above experimental groups. ***P < 0.001 versus the control group; ^###^*P* < 0.001 versus the IL-1β group; ^^^^^*P* < 0.001 versus the IL-1β+si-circSOD2 group. **D** The protein expression level of Aggrecan, MMP-13, and Collagen II were evaluated by Western blot in the above experimental groups. **E** The concentrations of IL-6, IL-8, and TNF-α were assessed by ELISA in the cell culture supernatant of the above experimental groups. ***P < 0.001 versus the control group; ^###^*P* < 0.001 versus the IL-1β group; ^^^*P* < 0.05, ^^^^*P* < 0.01, ^^^^^*P* < 0.001 versus the IL-1β+si-circSOD2 group

We further analyzed PRDX3 expression in CHON-001 cells transfected with si-NC, si-circSOD2, si-circSOD2+miR-224-5p inhibitor or si-circSOD2+PRDX3 expression vector in the presence of IL-1β challenge. IL-1β treatment elevated PRDX3 level, which was reduced by si-circSOD2. However, the co-transfection of miR-224-5p inhibitor or PRDX3 expression vector increased PRDX3 level upon circSOD2 silencing (Fig. 5A). In the above cell groups, cell proliferation assay revealed that after IL-1β treatment, the proliferation of CHON-001 cells was significantly inhibited, and si-circSOD2 rescued cell proliferation, while the co-transfection with PRDX3 vector or miR-224-5p inhibitor attenuated the rescue effect of si-circSOD2 (Fig. 5B). Similarly, flow cytometric analysis suggested that si-circSOD2 decreased the apoptosis rate in IL-1β-induced CHON-001 cells, while PRDX3 overexpression and miR-224-5p inhibitor further increased apoptotic rate in si-circSOD2 transfected cells (Fig. 5C). In addition, Western blot results suggested that the co-transfection with pcDNA-PRDX3 vector or miR-224-5p suppressor also impaired the effect of si-circSOD2 on ECM protein expression in IL-1β-treated CHON-001 cells (Fig. 5D). Furthermore, ELISA was adopted for detecting the levels of IL-6, IL-8, and TNF-α in CHON-001 cell supernatant, which revealed that the anti-inflammatory effect of circSOD2 knockdown was abrogated by the co-transfection with pcDNA-PRDX3 vector or miR-224-5p inhibitor (Fig. 5E). As indicated by these findings, circSOD2 mediates IL-1β-triggered damages in chondrocytes through the miR-224-5p/PRDX3 axis.

## Discussion

Current treatment goals for OA are mainly to alleviate the pain and improve joint functionality so as to upgrade the quality of life in the patients [21]. Chondrocytes, the functional cellular components present in the cartilage, are capable of undergoing a dynamic proliferation-apoptosis equilibrium, which maintains the biosynthetic and degradation cycles of the cartilage tissues under normal physiological conditions [22]. In the present study, the cartilage tissues of the knee joint from 25 OA patients were collected, and IL-1β, a pivotal inflammation mediator which exerts a crucial action on the inflammatory damages of OA [23], was used to treat chondrocytes to mimic OA-associated damages. The production of IL-1β is involved in diverse cellular events, including cell proliferation and apoptosis. The elevated IL-1β production is linked to the tissue injury and can reflect inflammatory severity [24]. In OA patients, IL-1β has been recognized as a major inducer of cartilage injury [25]. IL-1β triggered inflammatory responses and the degradation of cartilage tissues by matrix metalloproteinases (MMPs), cyclooxygenase-2 (COX-2) and inducible nitric oxide synthase (iNOS) [26].

CircRNAs are a class of circular non-coding RNAs and shows tissue-specific expression patterns, which attracts increasing research attention in recent years [27]. The dysregulation of circRNAs has been implicated in OA progression, including the proliferation and apoptosis in chondrocytes. In our study, we reported a new circRNA circSOD2 and its implication in OA progression. Further, we confirmed the interaction between circSOD2 and miR-224-5p, and between miR-224-5p and PRDX3, which overall suggests that circSOD2 regulates chondrocyte activity through the miR-224-5p/PRDX3 pathway in OA.

Based on the public circRNA profiling data in the cartilage tissues of the OA patients and healthy controls, circSOD2 was identified as a top upregulated circRNA in the OA samples. This observation was verified in the tissues of 25 patients with OA, as well as in IL-1β induced chondrocytes. Following the determination of circSOD2 as our target, loss-of-function experiments were performed to show the protective effect of circSOD2 knockdown in chondrocyte cell model. The knockdown of circSOD2 in chondrocyte ameliorated the detrimental effects of IL-1β on ECM-related proteins (MMP13, Aggrecan and Collagen II), apoptosis, and inflammatory responses. Competing endogenous RNA (ceRNA) networks have been shown to involve various non-coding RNAs to regulate the progressions of many disorders, including OA [28]. For example, circTMBIM6 promotes the OA-triggered ECM degradation through the miR-27a/MMP13 pathway [29]. Moreover, circ-UBE2G1 aggravates chondrocyte injury induced by lipopolysaccharide (LPS) via mediating the miR-373/HIF-1a pathway [30]. In this work, we demonstrated the functional interaction between circSOD2 and miR-224-5p. miR-224-5p was downregulated in OA samples and IL-1β-induced chondrocyte. The application of miR-224-5p inhibitor could impair the protective effect of circSOD2 silencing. Therefore, miR-224-5p mediates the protective effect of circSOD2 silencing in IL-1β-induced chondrocyte damages.

We further revealed PRDX3 as the mRNA target of miR-224-5p. miR-224-5p negatively regulates PRDX3 expression in chondrocytes, and silencing circSOD2 reduced PRDX3 expression. Therefore, we propose that circSOD2 serves as a miR-224-5p sponge to repress PRDX3 expression in chondrocyte. StarBase analysis showed the presence of miR-224-5p binding sites at the 3′ untranslated region of PRDX3 mRNA, and PRDX3-miR-224-5p interaction was validated via the luciferase reporter assay. We further analyzed the expression of PRDX3 in chondrocytes and found the enhanced level of PRDX3 following the IL-1β treatment, which could be reduced by si-circSOD2 or miR-224-5p mimic. Besides, results of proliferation assay, apoptosis detection and the analysis of inflammatory cytokines all suggest that miR-224-5p/PRDX3 axis mediates the effect of circSOD2 IL-1β-induced chondrocyte damages. Collectively, these data indicate that the overexpression of circSOD2 aggravates the progression of OA by repressing miR-224-5p activity and elevating PRDX3 expression.

There are several points needing to be further clarified in the future experiments. The mechanism underlying circSOD2 upregulation in OA requires further studies, which may provide novel insights into the formulation of intervention strategies. Further, how PRDX3 impinges on chondrocyte damages and its role in the animal model of OA warrant future investigations.

## Conclusion

In conclusion, circSOD2 is a circRNA showing overexpression during the progression of OA. The knockdown of circSOD2 ameliorates apoptosis, inflammatory response, and ECM degradation in the cell model of OA. The protective effect of circSOD2 silencing was possibly executed through modulating the miR-224-5p/PRDX3 signaling pathway. Our finding provided a possible therapeutic target for OA management.

## Supplementary Information

**Additional file 1. Fig. S1**: qRT-PCR analysis was performed to confirm the expression of the OA-related circRNAs identified in GSE175959 dataset using the cartilage samples from OA patients and controls.

**Additional file 2: Fig. S2**: Starbase was employed to predict the mRNA candidates of miR-224-5p. The expression level of these mRNA candidates were measured by qRT-PCR in CHON-001 cells upon the transfection of miR-224-5p mimic or miR-NC. ***P < 0.001 compared to miR-NC group.

## Data Availability

The data are available upon reasonable request.

## Abbreviations

OA: Osteoarthritis
ECM: Extracellular matrix
circRNAs: Circular RNAs
miRNA: MicroRNA
qRT-PCR: Reverse transcription-quantitative
OARSI: Osteoarthritis Research Society International
GEO: Gene Expression Omnibus
CHON-001: Human chondrocyte cell line
ELISA: Enzyme-linked immunosorbent assay
RIP: RNA immunoprecipitation
SDS–PAGE: Sodium dodecyl sulfate polyacrylamide gel electrophoresis
PVDF: Polyvinylidene difluoride
H&E: Hematoxylin and eosin
MMPs: Matrix metalloproteinases
iNOS: Inducible nitric oxide synthase
COX-2: Cyclooxygenase-2
SD: Standard deviation
