# Supplementary figures and images for: Knockdown of circSOD2 ameliorates osteoarthritis progression via the miR-224-5p/PRDX3 axis
Source: J Orthop Surg Res. 2023 Jun 13;18:432. doi: 10.1186/s13018-023-03880-9 (PMC10265860; doi:10.1186/s13018-023-03880-9)

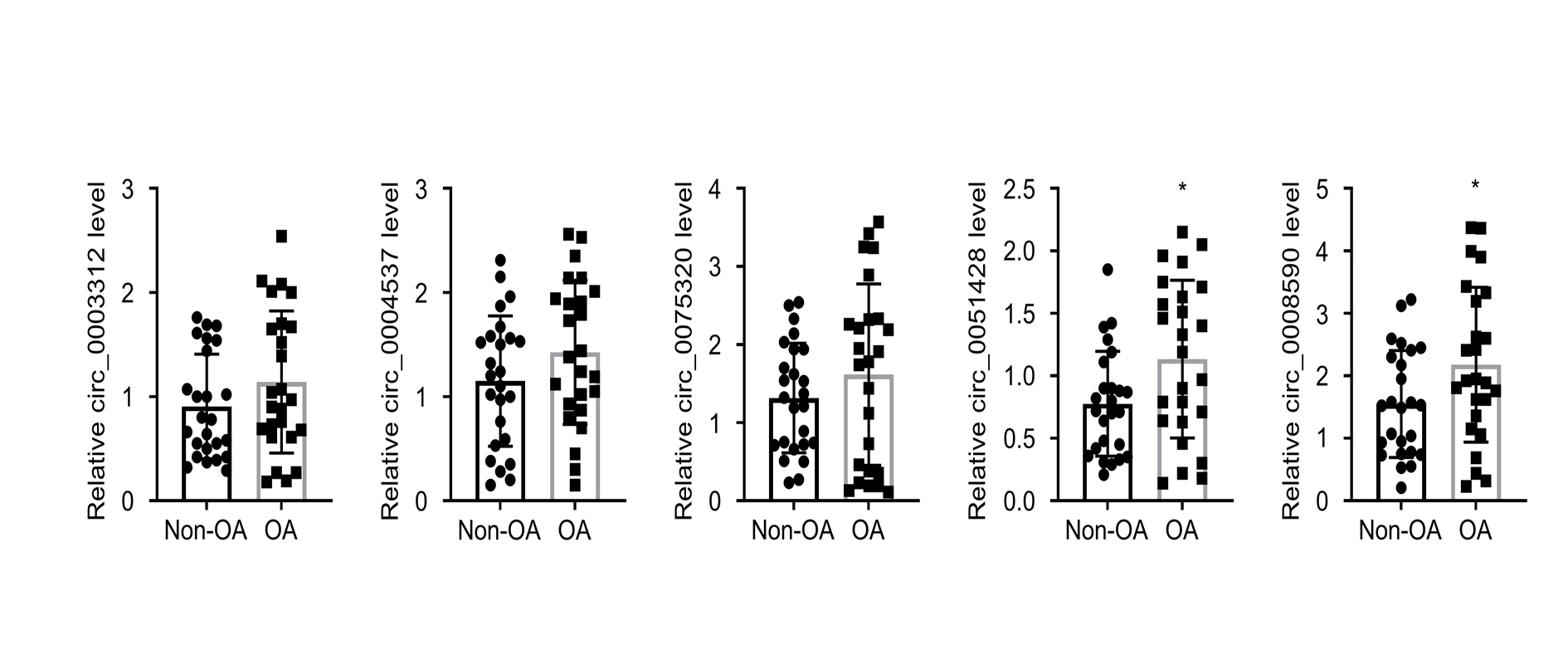

Supplement: Supplementary file 1 — Additional file 1. Fig. S1: qRT-PCR analysis was performed to confirm the expression of the OA-related circRNAs identified in GSE175959 dataset using the cartilage samples from OA patients and controls. [file 13018_2023_3880_MOESM1_ESM.jpg]

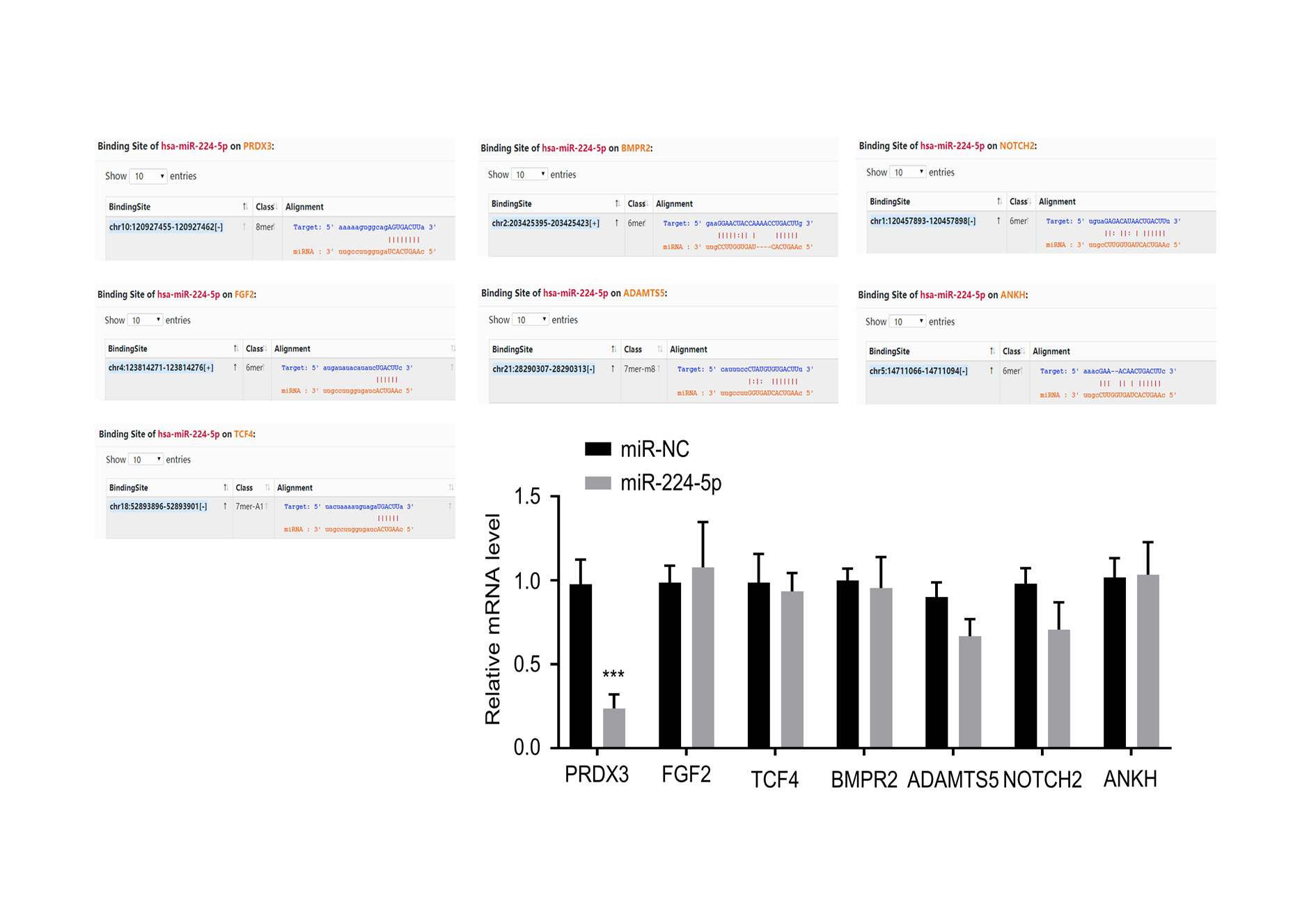

Supplement: Supplementary file 2 — Additional file 2: Fig. S2: Starbase was employed to predict the mRNA candidates of miR-224-5p. The expression level of these mRNA candidates were measured by qRT-PCR in CHON-001 cells upon the transfection of miR-224-5p mimic or miR-NC. ***P < 0.001 compared to miR-NC group. [file 13018_2023_3880_MOESM2_ESM.jpg]
